# The First Genome-Wide Survey Analysis of the Tibetan Plateau Tetraploid *Schizothorax curvilabiatus* Reveals Its Microsatellite Characteristics and Phylogenetic Relationships

**DOI:** 10.3390/genes16050491

**Published:** 2025-04-25

**Authors:** Bingjian Liu, Luxiu Gao, Yifan Liu, Kai He, Hongchi Li, Taobo Feng, Mingzhe Han, Chi Zhang

**Affiliations:** 1 National Engineering Research Center for Marine Aquaculture, Zhejiang Ocean University, Zhoushan 316022, China; bjetbj@163.com (B.L.); gaoluxiu@zjou.edu.cn (L.G.); et999927@163.com (Y.L.); fengtaobo@zjou.edu.cn (T.F.); hanmingzhe@zjou.edu.cn (M.H.); 2 National Engineering Laboratory of Marine Germplasm Resources Exploration and Utilization, Zhejiang Ocean University, Zhoushan 316022, China; 3 Institute of Fisheries Science, Tibet Academy of Agricultural and Animal Husbandry Sciences, Lhasa 850000, China; 17803296221@163.com (K.H.); lhd19940702@163.com (H.L.)

**Keywords:** *Schizothorax curvilabiatus*, genome survey, molecular markers, repetitive sequences, phylogenetic

## Abstract

**Background/Objectives**: *Schizothorax curvilabiatus*, a typical highland polyploid species within the subfamily Schizothoracinae, holds economic value and ecological research significance. Currently, there are no related genomic studies. To obtain its genetic information and lay the foundation for subsequent whole-genome map construction, this study conducted a genome survey analysis, preliminary genome assembly, microsatellite identification, repeat sequence annotation, mitochondrial genome characterization, and phylogenetic relationship research. **Methods**: DNA was sequenced on a DNBSEQ-T7 platform to obtain paired-end genomic data. The genome was analyzed using GCE, and the draft genome was assembled with SOAPdenovo. Microsatellites were identified using MISA, and the mitochondrial genome was assembled with NOVOPlasty. Genome features were analyzed, and phylogenetic trees were constructed using PhyloSuite and MEGA. **Results**: The genome size was estimated at 2.53 Gb, with a heterozygosity of 6.55% and 47.66% repeat sequences. A 1.324 Gb preliminary genome draft was obtained, with repeat sequences comprising 47.17%, the majority being DNA transposons (24.64%). Dinucleotide repeats were most abundant (46.91%), followed by mononucleotide repeats (38.31%), with A/T and AC/GT being the most frequent. A complete mitochondrial genome of 16,589 bp was assembled, and a 939 bp D-loop was annotated. Phylogenetic relationships among genera in the Schizothoracinae subfamily were also clarified. **Conclusions**: This study provides the latest molecular data for analysis of the *S. curvilabiatus* genome and its related populations, and for the first time offers genomic resources for research on genomic adaptive evolution and polyploidization in high-altitude environments.

## 1. Introduction

*Schizothorax curvilabiatus* (Cypriniformes: Cyprinidae: Schizothoracinae: *Schizothorax*) is a Tibetan Plateau–endemic fish species primarily distributed in the middle and lower reaches of the Yarlung Tsangpo River (e.g., Motuo and Yigong Lake) and its tributaries, including the Chayu River and Danba Qu in Tibet, China. It is typically found in nearshore rapids or river forks [1]. As a highland species, *S. curvilabiatus* exhibits distinctive biological traits such as slow growth, relatively long lifespan, late sexual maturity, and low fecundity [1,2,3], and is particularly sensitive to overexploitation of riverine resources and environmental degradation [4]. Recognized for its dual ecological and economic significance, this species not only plays a crucial role in maintaining river ecosystem stability, but also holds potential value for aquaculture development.

The advancement of next-generation sequencing (NGS) technologies has enabled the sequencing of genomes for an increasing number of species, including animals [5], plants [6], and fungi [7]. According to data published in the NCBI (National Center for Biotechnology Information) and IGSR (International Genome Sample Resource) databases, as well as related studies, approximately 100,000 species have been sequenced to date, including over 2000 fish species encompassing model organisms and commercially important varieties. As a polyploid species endemic to high-altitude environments, *S. curvilabiatus* likely exhibits unique genomic traits such as duplicated gene retention, repeat element expansion, and chromosomal restructuring, which may underpin its adaptation to extreme conditions like hypoxia and temperature fluctuations. However, the impact of polyploidization on its genome organization remains unexplored, limiting our understanding of its evolutionary mechanisms and conservation strategies.

Genome survey analysis based on NGS technologies, employing K-mer analysis to estimate genome size, heterozygosity, and GC content, serves as an effective approach for elucidating the genetic characteristics of organisms. These findings provide critical data for designing subsequent library construction and high-quality genome assembly strategies, while also playing a critical role in the development of genome-wide microsatellite markers (simple sequence repeats, SSRs) [8]. Here, we conduct the first genome survey of *S. curvilabiatus* to characterize its polyploid genome architecture, including repeat elements, microsatellite distribution, and mitochondrial genome features, and to explore how polyploidy may shape genomic evolution within Schizothoracinae fishes.

*S. curvilabiatus* has been identified as a typical polyploid fish species endemic to high-altitude environments [9]. Conducting genome survey analysis on this species would provide critical insights into its genomic evolutionary characteristics and phylogenetic relationships, thereby serving as a reference for subsequent genetic studies and aquaculture applications. In recent years, due to habitat destruction, environmental pollution, and climate change, its population has declined significantly, leading to its classification as a priority aquatic species for conservation. Current research on *S. curvilabiatus* has been limited to preliminary descriptions of growth classification, nutritional composition, and habitat-related dietary habits [10,11,12,13,14]. Furthermore, the sole published mitochondrial genome, which lacks comprehensive analysis, dates back to an earlier period [15], leaving the *S. curvilabiatus* genome largely unexplored.

Simple sequence repeats (SSRs), also referred to as microsatellites or minisatellites, are hypervariable DNA motifs composed of tandem repeats of short nucleotide patterns, exhibiting mutation rates approximately 10-fold higher than single-nucleotide polymorphisms [16]. SSRs are uniformly distributed across eukaryotic genomes and have been widely applied in studies of population genetic diversity and kinship identification, as well as in genetic map construction [17,18]. In polyploid genomes, SSRs may exhibit distinct distribution patterns due to genome duplication–induced redundancy, potentially affecting functional evolution and adaptability. Investigating SSRs in *S. curvilabiatus* could therefore reveal polyploidy-specific genomic signatures and adaptation mechanisms. Compared to conventional SSRs development workflows, SSRs mining based on high-throughput sequencing data offers advantages of low cost, high efficiency, and enhanced throughput. Furthermore, SSRs are hypothesized to serve as critical functional elements facilitating evolutionary adaptation and rapid environmental responsiveness [19]. However, the genomic distribution and characteristics of SSRs in *S. curvilabiatus* remain poorly understood.

This study aimed to address critical gaps in understanding the genome of *S. curvilabiatus* by conducting the first genome survey to systematically characterize its polyploid genome architecture, including repeat element dynamics, microsatellite (SSR) distribution patterns, and mitochondrial genome features. We further investigate the evolutionary mechanisms through which polyploidy may underpin its adaptation to extreme high-altitude environment. By developing a comprehensive SSR marker suite, this work seeks to support future population genetic studies and conservation management strategies. Additionally, the foundational genomic data generated herein will inform both evolutionary hypotheses and practical frameworks for the development of plateau-adapted aquaculture practices.

## 2. Materials and Methods

### 2.1. Sample Collection and DNA Extraction

Muscle tissue samples were taken from *S. curvilabiatus* collected from the Chayu River (97°02′49.81″ E, 28°27′20.56″ N) in Zhuwagen Town, Chayu County, Tibet, China, in January 2024. Genomic DNA was extracted from the muscle tissue using a standard phenol-chloroform method [20]. DNA concentration and integrity were assessed using a Pultton DNA/Protein Analyzer (Plexech) and 1% agarose gel electrophoresis, respectively. The muscle tissue specimens were deposited in the Laboratory of Marine Germplasm Resource Exploration and Utilization, School of Marine Science and Technology, Zhejiang Ocean University, under the voucher number CY2024001.

### 2.2. Sequencing and Data Quality Control

Paired-end whole-genome libraries were constructed from quality-checked DNA samples using an MGIEasy FS DNA Prep Kit (BGI, Shenzhen, China) according to the standard workflow of the DNBSEQ-T7 platform (BGI), with an insert size of 350 bp. Sequencing was performed on the DNBSEQ-T7 platform following library quality verification. Raw sequencing data were processed using Fastp v 0.24.0 [21] and FastQC v0.11.5 [22] to remove adapter sequences and low-quality reads. The raw data were deposited in the Sequence Read Archive (SRA) database (http://www.ncbi.nlm.nih.gov/sra/) under the accession number SRR32816543. Sequencing services were provided by Xi’an Haorui Gene Technology Co., Ltd. Xi’an, China.

### 2.3. Genome Evaluation and De Novo Assembly

Sequencing data from *S. curvilabiatus* were analyzed using GCE v1.0.2 [23] to estimate genome size, GC content, and heterozygosity. A K-mer distribution analysis was performed by simulating K-mer frequencies across the genome under a Poisson distribution model with the recommended parameter K = 17. The depth and total count of K-mers were utilized for genome characterization. De novo genome assembly was performed using SOAPdenovo2 vr242 [24] based on the effective sequencing data, with Contig and Scaffold sizes calculated (K = 17).

### 2.4. Microsatellite Identification and Repeat Sequence Annotation

Following genome assembly, SSRs loci were identified from the sequences using the MISA script with default parameters (minimum repeat thresholds for mono- to hexanucleotide motifs: 10, 6, 5, 5, 5, and 5, respectively). SSRs primers were batch-designed using Primer3 v 2.6.0 software [25] and subsequently synthesized by Sangon Biotech (Shanghai) Co., Ltd., Shanghai, China. The validity of the primers and SSRs was verified through polymerase chain reaction (PCR) amplification followed by agarose gel electrophoresis analysis.

Two approaches were employed to annotate repetitive sequences in the *S. curvilabiatus* genome: homology prediction and de novo prediction. For homology prediction, the genome sequences were first partitioned by chromosome using the para_RepeatMasker script provided by GETA v2.5.7 (https://github.com/chenlianfu/geta). Parallelized RepeatMasker v4.1.7-p1 [26] was then executed for homology repeat annotation, with the reference species (--RM_species) specified as Cypriniformes and the Repbase database utilized as the reference. For de novo prediction, a custom repeat database was constructed using the BuildDatabase program in RepeatModeler v2.0.5 [27] and BLAST v2.15.0 [28] to generate database indices. RepeatModeler was subsequently run to identify species-specific repetitive elements. Finally, repeat annotations from both methods were merged using the merge_repeatMasker_out.pl script

### 2.5. Genomic Features and Phylogenetic Analysis

The complete mitochondrial genome of *S. curvilabiatus* was assembled using NOVOPlasty v4.3.5 [29] with paired-end sequencing data and the previously published mitochondrial genome (GenBank accession no. NC_035994) as a reference. The assembled mitochondrial genome was annotated using the MITOS2 web server [30] (http://mitos2.bioinf.uni-leipzig.de/index.py), followed by manual curation of the annotation results in BioEdit v7.2.6 [31]. A mitochondrial genome map was generated using the CGView Server [32]. Genomic features, including nucleotide composition, base bias (AT skew = [A − T]/[A + T]; GC skew = [G − C]/[G + C]), amino acid composition, and relative synonymous codon usage (RSCU), were analyzed using PhyloSuite v1.2.0 [33]. tRNA genes were predicted and visualized using tRNAscan-SE v2.0 [34]. The complete mitochondrial genome sequence was deposited in the NCBI GenBank database under accession number PV287704.

Mitochondrial genome sequences of 23 species spanning 14 genera within the subfamily Schizothoracinae were retrieved from the NCBI GenBank database (https://www.ncbi.nlm.nih.gov/genbank/) for phylogenetic analysis with the study species (Table 1). Thirteen protein-coding genes (PCGs) were aligned using MAFFT v7.520 [35], followed by trimming of ambiguous regions with Gblocks v0.91b [36]. Phylogenetic trees were reconstructed using two approaches: (a) Bayesian inference (BI)**,** implemented in PhyloSuit v1.2.0 [33] under the JC+I+G model, with Markov Chain Monte Carlo (MCMC) parameters set to 600,000 generations; and (b) maximum likelihood (ML), conducted in MEGA11 [37] under the GTR+G+I model, with 1000 bootstrap replicates. The resulting phylogenetic trees were visually refined using the iTOL web server (https://itol.embl.de/) [38].

## 3. Results

### 3.1. Genome Sequencing, K-Mer Analysis, and De Novo Assembly

**Figure 1:**
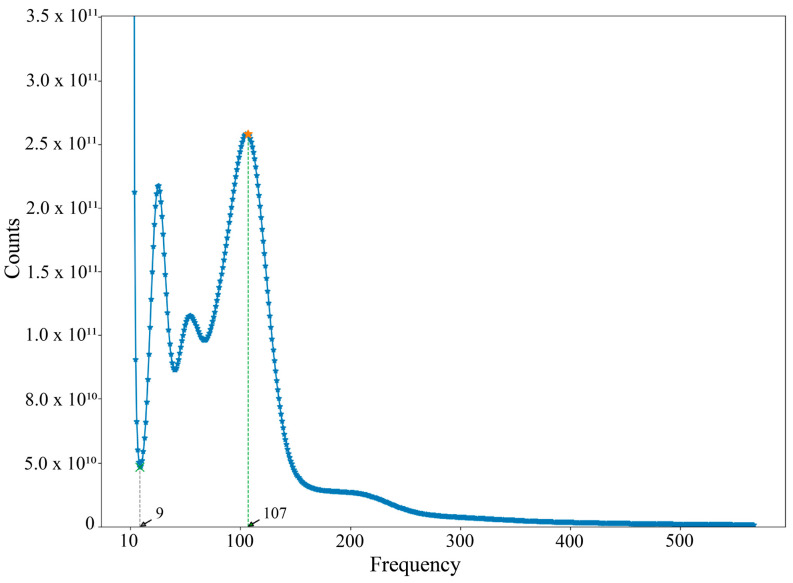
Depth profile of K-mer (17-mer) distribution in the *S. curvilabiatus* genome. The first peak (frequency=9) is usually in the region of lower frequency caused by sequencing errors at low frequencies K-mer, while the main peak (frequency = 107 in the higher frequency region) usually corresponds to the true genome sequence. It can be expressed as the average depth of coverage of sequencing.

High-throughput sequencing on the DNBSEQ-T7 platform generated 117.71 Gb of raw data, with 109.48 Gb of high-quality data retained after quality control. The GC content, Q20, and Q30 values were 38.38%, 98.88%, and 96.19%, respectively (Table 2), indicating robust sequencing quality. K-mer analysis (k = 17) yielded a total of 270,100,730,872 K-mers. Based on the average coverage depth (107×), the genome size of *S. curvilabiatus* was estimated as approximately 2.53 Gb, with a heterozygosity of 6.55% and a repetitive sequence content of 47.66% (Figure 1), consistent with its status as a highly heterozygous polyploid species. De novo assembly produced a genome draft of 1,743,555,986 bp in contigs and 1,841,773,848 bp in scaffolds, with contig N50 and scaffold N50 values of 33,205 bp and 33,343 bp, respectively. The overall GC content of the assembled genome was 37.57% (Table 3).

### 3.2. Genomic SSR Distribution

**Figure 2:**
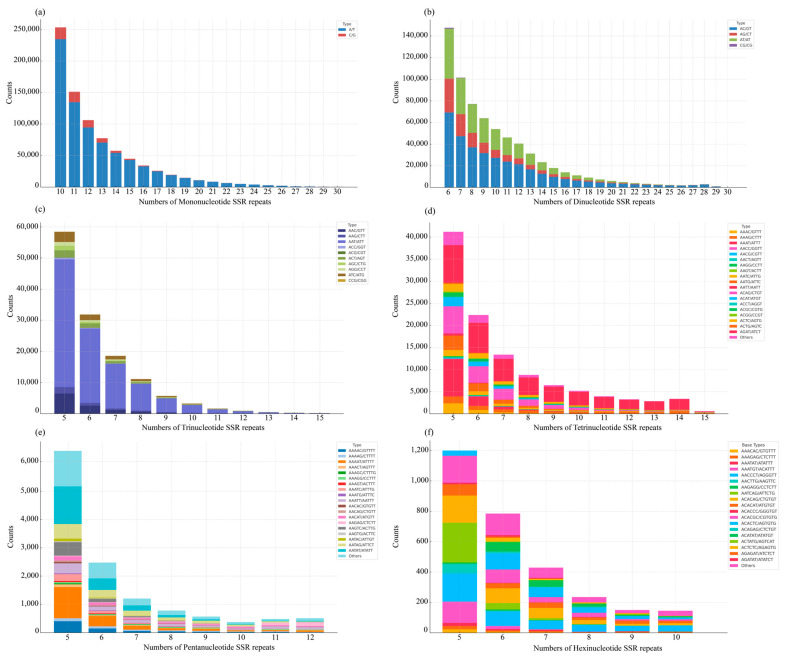
Frequency distribution of microsatellite loci in *S. curvilabiatus*. (**a**) Mononucleotides; (**b**) dinucleotides; (**c**) trinucleotides; (**d**) tetranucleotides; (**e**) pentanucleotides; (**f**) hexanucleotides.

A total of 1,768,499 SSRs loci were identified in the de novo assembly of the *S. curvilabiatus* genome. Dinucleotide repeats accounted for the highest proportion (46.91%, 829,582), followed by mononucleotide (38.31%, 677,463), trinucleotide (7.52%, 132,881), tetranucleotide (6.31%, 111,590), pentanucleotide (0.77%, 13,159), and hexanucleotide repeats (0.22%, 3824). The SSRs motifs encompassed four distinct mononucleotide types, 12 dinucleotide types, 60 trinucleotide types, 230 tetranucleotide types, 676 pentanucleotide types, and 628 hexanucleotide types. The most frequent motifs were A/T for mononucleotides, AC/GT for dinucleotides, AAT/ATT for trinucleotides, AGAT/ATCT for tetranucleotides, AATAT/ATATT for pentanucleotides, and AACCCT/AGGGTT for hexanucleotides (Figure 2).

### 3.3. Genomic Repeat Sequence Annotation

Repeat sequences in the *S. curvilabiatus* genome assembly were annotated through homology and de novo approaches, identifying 868,837,324 bp of repetitive sequences, accounting for 47.17% of the genome (Table 4). Non-interspersed repeats comprised simple repeats (3.28%), satellite sequences (1.89%), and low-complexity regions (0.45%). Among interspersed repeats, DNA transposons constituted the largest proportion (24.64%), followed by long terminal repeats (LTRs, 3.04%), long interspersed nuclear elements (LINEs, 2.47%), short interspersed nuclear elements (SINEs, 0.56%), and retroposons (0.02%).

### 3.4. Mitochondrial Genome Structural Features

**Figure 3:**
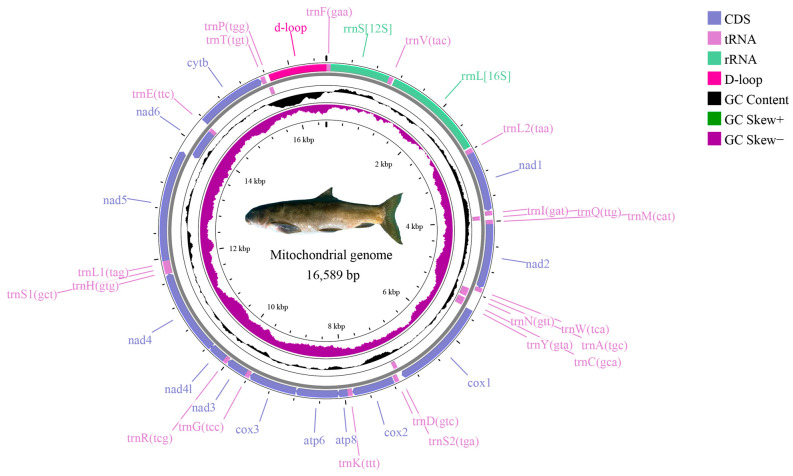
Mitochondrial genome structure of *S. curvilabiatus*.

The complete mitochondrial genome of *S. curvilabiatus* exhibited a typical double-stranded closed circular structure with a total length of 16,589 bp, comprising 22 tRNAs, 2 rRNAs, 13 protein-coding genes (PCGs), and one non-coding control region (D-Loop) (Figure 3). Among these 37 genes, *trnQ*, *trnA*, *trnN*, *trnC*, *trnY*, *trnS2*, *nad6*, *trnE*, and *trnP* were located on the light strand (L-strand), while the remaining genes resided on the heavy strand (H-strand). Gene arrangements included intergenic spacers, overlapping regions, and non-coding sequences. Ten overlapping regions (total length: 28 bp) and 16 intergenic spacers (total length: 117 bp) were identified. The longest overlaps (7 bp) occurred between *atp8* and *atp6* and between *nad4L* and *nad4*. The largest intergenic spacer (33 bp) was situated between *trnN* and *trnC* (Table 5). The mitochondrial genome displayed nucleotide compositions of 30.1% A, 25.4% T, 27.1% G, and 17.5% C. The overall A+T content of the 13 PCGs was 54.3%, with *atp8* exhibiting the highest A+T content (60.6%). The calculated AT skew (0.085) and GC skew (−0.214) indicated a higher mutation rate in A/C bases compared to G/T bases (Table 6).

**Figure 4:**
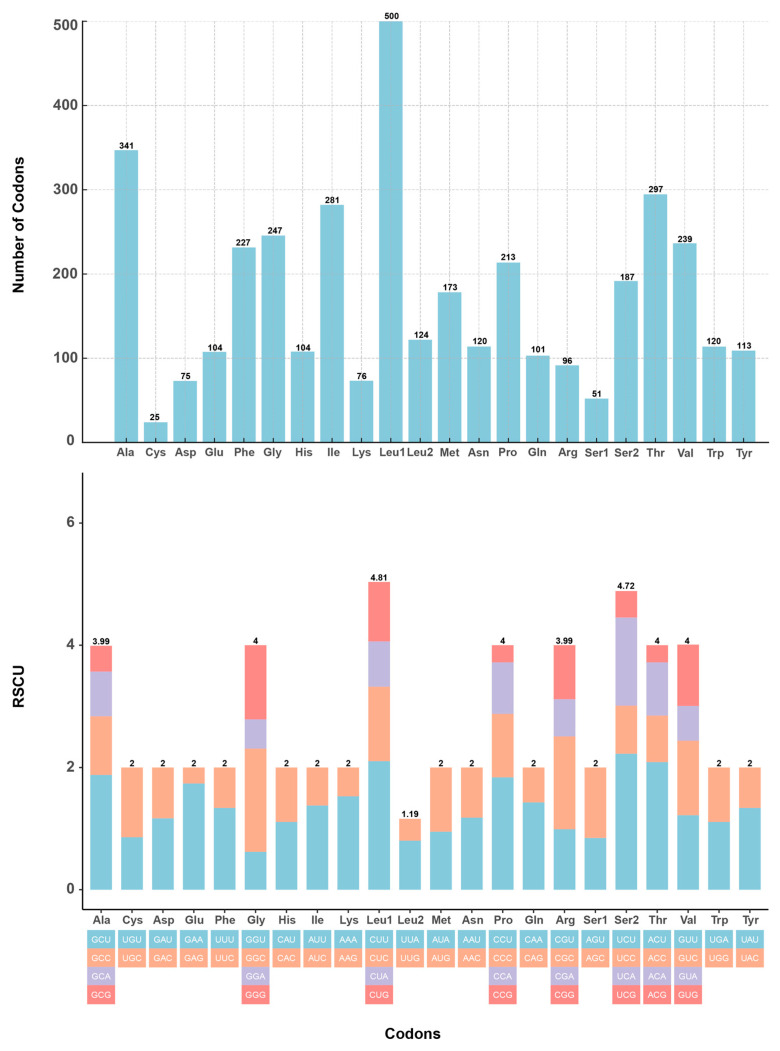
Amino acid composition and RSCU usage in the mitochondrial genome of *S. curvilabiatus*.

**Figure 5:**
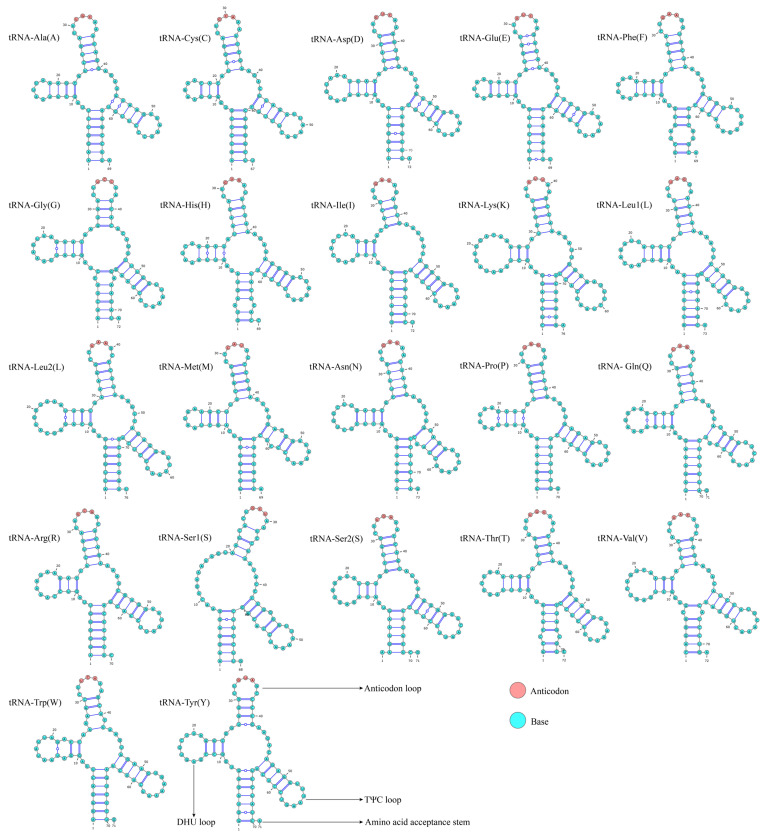
Secondary structure of tRNAs in the mitochondrial genome of *S. curvilabiatus*.

In this study, the mitochondrial genome of the *S. curvilabiatus* encoded a total of 3794 amino acids. Among them, *trnLeu1* (500, 13.13%) and *trnAla* (341, 8.98%) exhibited higher usage frequencies, while *trnCys* (25, 0.65%) and *trnSer1* (51, 1.34%) had lower frequencies. Additionally, the usage of synonymous codons showed a distinct bias for most amino acids. A total of 30 codons exhibited a relative synonymous codon usage (RSCU) value ≥1, with the codons CUA (*trnLeu1*), CCA (*trnPro*), and CGA (*trnArg*) having RSCU values greater than 2 (Figure 4). In terms of secondary structure, all 21 tRNAs of *S. curvilabiatus*, except for *trnSer1*, were able to fold into a typical cloverleaf structure. Besides the normal base pairings, base mismatches between G and U were also observed (Figure 5).

### 3.5. Phylogenetic Relationships of S. curvilabiatus

**Figure 6:**
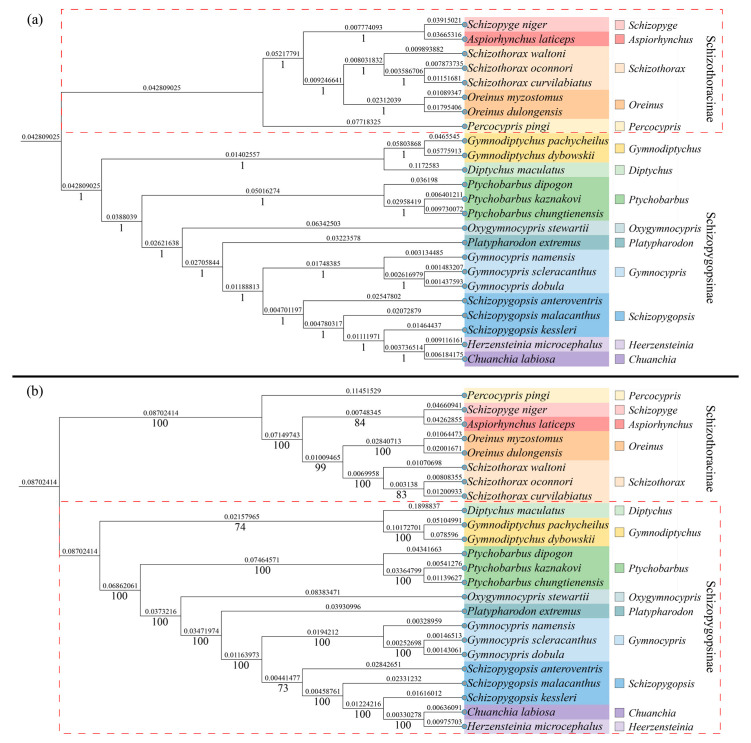
Phylogenetic tree of *S. curvilabiatus* based on 13 PCGs. Note: (**a**) BI; (**b**) ML.

Phylogenetic trees were constructed for 14 genera within Schizothoracinae and Schizopygopsinae using both BI and ML methods. Both trees exhibited consistent topological structures (Figure 6). Within Schizothoracinae, the genera *Schizothorax*, *Oreinus*, *Percocypris*, *Schizopyge*, and *Aspiorhynchus* formed a monophyletic group, while the remaining genera, including *Schizopygopsis*, *Ptychobarbus*, *Gymnocypris*, and *Gymnodiptychus*, clustered into another branch. Additionally, *Schizothorax* and *Oreinus,* as well as *Gymnodiptychus* and *Diptychus,* formed smaller subgroups. Within *Schizothorax*, the relationship between *S. curvilabiatus* and *S. oconnori* was the closest. The most closely related genera between the two subfamilies were *Heerzensteinia* and *Chuanchia*.

## 4. Discussion

The emergence of sequencing technologies led to completion of the first fish genome sequence (*Takifugu rubripes*) in 2002, marking the beginning of a new era in fish genomics research [39]. Currently, more than 31,000 species of fish inhabit the Earth, including teleosts, jawless fish, and cartilaginous fish, with several species holding significant ecological and economic value. With the launch of initiatives such as the “Chinese Aquatic 10-100-1000 Genomics Program” and the “Fish 10K Project” [40,41], the number of fish genomes available has increased significantly. Notable species include model organisms like *Danio rerio* [42] and *Oryzias sinensis* [43], economically important species such as *Cyprinus carpio* [44] and *Ctenopharyngodon idella* [45], and ecologically valuable species like the cave fish *Astyanax mexicanus* [46] and the high-altitude fish *S. younghusbandi* [47]. However, as of now, the fish genomes published in the NCBI database (https://www.ncbi.nlm.nih.gov/datasets/genome/, assessed on 24 March 2025) account for less than 10% of the global fish species (2832 species).

In this study, a new genome (*S. curvilabiatus*) was sequenced. *S. curvilabiatus*, a flagship polyploid species of the Schizothoracinae subfamily and the *Schizothorax* genus, is an important economic species in Tibet, China and is regarded as an ideal model for understanding the “evolution-geology synergistic mechanism” (ascent with modification) [48,49]. However, only 3 out of the 45 species in the *Schizothorax* genus have had their genomes sequenced to date. Based on K-mer analysis, we estimate that the genome size of *S. curvilabiatus* is approximately 2.53 Gb, with the preliminary assembly resulting in a genome size of about 1.84 Gb (Table 3). Compared to previously published genomes of *Schizothorax* species, the draft genome of *S. curvilabiatus* is smaller than that of *S. oconnori* (~2.07 Gb) [50] but larger than that of *S. macropogon* (~1.56 Gb) [51] and *S. lantsangensis* (972.4 Mb). It is similar in size to that of *O. stewartii* (~1.85 Gb) [52]. We hypothesize that the variation in genome size among *Schizothorax* species may be due to the accumulation of repetitive elements during species genome evolution and the limitations of short-read data from next-generation sequencing. In future studies, long-read sequencing technologies, such as Single Molecule Real-Time (PacBio SMRT), combined with Hi-C (High-throughput chromosome conformation capture) technology, can be used to construct high-quality genome maps. The observed genome size variation (1.56–2.53 Gb) among *Schizothorax* species may also reflect polyploidy-driven genomic dynamics. Polyploidization events (e.g., whole-genome duplication) typically induce genome expansion through the retention of duplicated chromosomal regions and repetitive element proliferation. For instance, the larger genome size of *S. curvilabiatus* compared to *S. lantsangensis* (972.4 Mb) could have resulted from differential diploidization rates post-polyploidization, where some species rapidly purge redundant sequences, while others retain them for adaptive potential [53]. Similar patterns have been documented in tetraploid carp (*C. carpio*), where genome duplication facilitates environmental adaptation by preserving stress-responsive gene duplicates [54]. The genome survey analysis conducted in this study provides preliminary insights into the genomic characteristics of *S. curvilabiatus* and lays the foundation for further whole-genome sequencing and assembly, significantly enriching the genomic resources of the *Schizothorax* genus.

SSRs are widely distributed in both prokaryotic and eukaryotic genomes and are considered efficient molecular markers for genetic differentiation and evolutionary studies due to their high efficiency and polymorphism [18,19]. In this study, 1,768,499 loci were identified in the *S. curvilabiatus* genome, with dinucleotides being the most abundant repeat sequence (46.91%). This finding is consistent with results from studies on *S. waltoni* [55], *S. biddulphi* [56,57], *S. prenanti* [58], *Gymnocypris eckloni*, and *G. przewalskii ganzihonensis* [59]. Additionally, the distribution patterns of nucleotide base repeats also align with these findings, with mononucleotide repeats predominantly consisting of A/T, dinucleotide repeats primarily composed of AC/GT, and trinucleotide repeats largely consisting of AAT/ATT. The complexity of repeat sequences often reflects the DNA mutation rate and evolutionary level of a species [60,61]. The distribution frequency from mononucleotides to trinucleotides was as high as 92.74%, indicating that *S. curvilabiatus* has accumulated significant genetic variation during its evolutionary process. The predominance of dinucleotide SSRs (46.91%) in *S. curvilabiatus* may reflect polyploidy-mediated genomic redundancy. In duplicated genomes, simpler repeat motifs (e.g., AC) are less likely to disrupt essential coding regions compared to complex trinucleotide repeats, allowing their accumulation under relaxed purifying selection. This pattern aligns with observations in other polyploid fish, such as the autotetraploid loach (*Misgurnus anguillicaudatus*), where dinucleotide SSRs dominate due to their tolerance to mutation in redundant genomic regions [62]. Furthermore, the high SSR polymorphism (1,768,499 loci) could enhance adaptive plasticity, a critical trait for survival in the fluctuating environments of the Tibetan Plateau. Based on these SSRs, this study provides valuable resources for future population genetics research.

Repetitive elements, particularly transposons, are critical components of eukaryotic genomes and serve as a source of genetic variation [63,64]. In the assembled genome draft of *S. curvilabiatus*, a total of 47.17% (868.84 Mb) of the sequence was identified as repetitive, with DNA transposons being the most prevalent type of repeat sequence (453.96 Mb, 24.64%) (Table 4). DNA transposons have also been identified as the dominant type of transposable elements (TEs) in other plateau species, such as *S. oconnori* [50], *Glyptosternon maculatum* [65], *Triplophysa dalaica* [66], *G. przewalskii* [67], and *Hucho bleekeri* [68], while non-LTRs, LINEs, and SINEs were found to be less prominent. The high proportion of DNA transposons (24.64%) in *S. curvilabiatus* may be a direct consequence of polyploidization. Genome duplication events provide “safe havens” for transposon activity, as redundant genomic regions buffer against deleterious insertions. In high-altitude *Schizothorax* species, transposon-mediated genomic plasticity could facilitate rapid adaptation to hypoxia and temperature extremes by rewiring stress-responsive gene networks [69]. This suggests that DNA transposons play a significant role in the genomic adaptation and evolutionary processes of plateau species.

Mitochondria, as essential energy factories within cells, are primarily responsible for generating most of the energy required for cellular biochemical reactions [70]. Studies have shown that the genetic material carried by mitochondria (mtDNA) plays multiple crucial roles throughout an organism’s lifecycle, involving vital biological processes such as regulation of lifespan, maintenance of reproductive capacity, response to metabolic stress, adaptation to environmental changes, and thermoregulation [71,72]. Additionally, mitochondrial DNA fragments have been proven to be effective molecular markers for phylogenetic and population genetic analyses [73]. In this study, a complete mitochondrial genome of *S. curvilabiatus* was assembled using nuclear genome sequences, with a total length of 16,589 bp. This length is consistent with that of other species within the *Schizothorax* genus, while showing slight variation when compared to other species in the Schizothoracinae subfamily (Table 1). Upon comparison, the complete mitochondrial genome assembled in this study is slightly longer than previously reported (16,589 bp vs. 16,578 bp) [15]. The overall nucleotide composition of A, T, G, C bases and the relatively high AT content are consistent with previous findings (Table 6), aligning with the typical base composition characteristics of most teleosts, reflecting a strong mutation tendency, functional regulation, and evolutionary adaptability [74]. Additionally, other structural features of the mitochondrial genome of *S. curvilabiatus*, such as synonymous codon usage frequency and tRNA secondary structures, are consistent with those of other species within the *Schizothorax* genus [75,76,77,78,79]. The D-loop region, known as the “control center” of the mitochondrial genome, directly influences the cell’s energy supply, genetic stability, and environmental response by regulating replication, transcription, mutation accumulation, and metabolic adaptation. Its high variability and functional importance make it a key target for mitochondrial biology, medicine, and evolutionary research [80,81]. In our study, a D-loop region of 939 bp was successfully annotated, which has not been previously reported in prior studies. Based on 13 protein-coding genes (PCGs), we provide a more systematic and comprehensive analysis of the phylogenetic relationships among species in the Schizothoracinae subfamily (Figure 6). Clear clustering differentiation was observed among genera, with *S. curvilabiatus* showing the closest phylogenetic relationship to *S. oconnori*, consistent with previous studies [15,82,83,84]. Studies have shown that phylogenetic relationships inferred from mitochondrial data may conflict with nuclear genomic divergence patterns [85]. Such incongruence could arise from hybridization events during polyploid speciation, where mitochondrial genomes (maternally inherited) and nuclear genomes (recombined from parental lineages) capture distinct evolutionary histories [86]. Similar mitochondrial–nuclear discordance is widespread in polyploid *Cyprinidae* species, such as the *Carassius auratus* complex, where hybrid origins and genome duplication generate conflicting phylogenetic signals [87]. Currently, the phylogenetic relationships of *S. curvilabiatus* and other Schizothorax species based on nuclear genomic data remain unclear, and whether such conflicts exist within this genus is unknown. Future studies should integrate nuclear gene trees with cytogenetic analyses (e.g., fluorescence in situ hybridization) to resolve the polyploidization history of the *Schizothorax* genus.

## 5. Conclusions

In summary, a genome survey analysis of the tetraploid species *S. curvilabiatus* from the Tibetan Plateau revealed a complex genome with relatively large genome size, high heterozygosity, and abundant repetitive sequences. A de novo genome draft was assembled, and its size is consistent with that of other species within the *Schizothorax* genus. Repetitive sequence annotation of the assembled genome revealed that TEs constitute a significant proportion of the genome, which may contribute to the genomic evolution of *S. curvilabiatus*, enabling it to better adapt to the plateau environment. Additionally, a large number of SSRs were identified, providing foundational information for the development of molecular markers. Furthermore, a complete mitochondrial genome was assembled from the sequencing data. A comprehensive analysis of its gene structure and codon usage was conducted, and the mitochondrial gene sequences obtained in this study were compared with the previously published mitochondrial genome of *S. curvilabiatus*. The results provide updated reference data for the mitochondrial genome analysis of *S. curvilabiatus* and its related population genetics studies. The phylogenetic tree based on the mitochondrial genome further clarifies the evolutionary positions of the genera within the Schizothoracinae subfamily, with a clearer understanding of the phylogenetic relationship of *S. curvilabiatus*. These findings provide genomic resources for the adaptive evolution and genome polyploidization of *S. curvilabiatus* in high-altitude environments and lay the foundation for constructing a high-quality genome map of *S. curvilabiatus* in future studies.

## Figures and Tables

**Table 1:** List of species and corresponding mitochondrial genomes with references.

Subfamily	Genus	Species	Accession No.	Length (bp)
Schizothoracinae	*Schizothorax*	*Schizothorax curvilabiatus*	PV287704	16,589
		*Schizothorax oconnori*	NC_020781.1	16,590
		*Schizothorax waltoni*	NC_020606.1	16,589
	*Oreinus*	*Oreinus myzostomus*	MW786773.1	16,578
		*Oreinus dulongensis*	NC_058201.1	16,579
	*Percocypris*	*Percocypris pingi*	NC_018601.1	16,586
	*Schizopyge*	*Schizopyge niger*	NC_022866.1	16,585
	*Aspiorhynchus*	*Aspiorhynchus laticeps*	NC_022855.1	16,591
Schizopygopsinae	*Schizopygopsis*	*Schizopygopsis anteroventris*	NC_029190.1	16,620
		*Schizopygopsis malacanthus*	NC_024880.1	16,677
		*Schizopygopsis kessleri*	NC_045935.1	16,767
	*Ptychobarbus*	*Ptychobarbus dipogon*	NC_024537.1	16,787
		*Ptychobarbus kaznakovi*	NC_025303.1	16,842
		*Ptychobarbus chungtienensis*	NC_034230.1	16,970
	*Gymnocypris*	*Gymnocypris namensis*	NC_021420.1	16,674
		*Gymnocypris scleracanthus*	NC_036349.1	16,679
		*Gymnocypris dobula*	NC_021419.1	16,720
	*Gymnodiptychus*	*Gymnodiptychus pachycheilus*	NC_023793.1	16,586
		*Gymnodiptychus dybowskii*	NC_028544.1	16,677
	*Diptychus*	*Diptychus maculatus*	NC_025650.1	16,859
	*Oxygymnocypris*	*Oxygymnocypris stewartii*	NC_022718.1	16,646
	*Platypharodon*	*Platypharodon extremus*	NC_029171.1	16,668
	*Heerzensteinia*	*Herzensteinia microcephalus*	NC_033403.1	16,726
	*Chuanchia*	*Chuanchia labiosa*	NC_029181.1	16,705

**Table 2:** Sequencing data statistics of *Schizothorax curvilabiatus*.

Data Type	Total Bases(G)	GC (%)	Q20 (%)	Q30 (%)
Raw data	117.71	38.37	98.91	96.28
Clean data	109.48	38.38	98.88	96.19

**Table 3:** De novo assembly of the *S. curvilabiatus* genome.

K = 17	Number	Total Size (bp)	GC (%)
Genome	/	/	37.57
Scaffold N50	/	33,343	/
Contig N50	/	33,205	/
Scaffolds	7,272,578	1,841,773,848	/
Contigs	8,853,863	1,743,555,986	/
Longest size	1	63,798	/
Sequences ≥ 1 kbp	213,379	384,572,145	/
Sequences ≥ 2 kbp	49,706	166,415,365	/
Sequences ≥ 3 kbp	20,556	96,556,207	/

**Table 4:** Landscape of repetitive elements in the de novo assembly of the *S. curvilabiatus* genome.

		Repeat Number	Repeat Size (bp)	Percentage (%)
Total		1,194,665	868,837,324	47.17%
Non-interspersed Repeats	Simple_repeat	230,353	70,680,192	3.83%
	Satellite	20,122	25,517,546	1.38%
	Low_complexity	30,473	10,538,499	0.57%
Interspersed Repeats	LTRs	24,203	56,086,412	3.04%
	LINEs	42,394	45,536,823	2.47%
	SINEs	17,390	10,472,053	0.56%
	Retroposon	814	485,054	0.02%
	DNA transposons	437,981	453,962,396	24.64%
Unknown		360,488	139,173,042	7.56%
Rolling-circles		6284	6,379,737	0.35%
rRNA		569	441,039	0.02%
tRNA		16,054	3,400,664	0.18%
snRNA		4153	899,668	0.05%
scRNA		3088	634,796	0.03%

**Table 5:** Features of the mitochondrial genome of *S. curvilabiatus*. H and L represent the heavy chain and light chains, respectively; T-- represents incomplete stop codons; – represents base overlap.

Gene	Position		Length (bp)	Codon		Intergenic Region	Strand
	From	To		Start	Stop		
*trnF*	1	69	69			0	H
*rrnS*	70	1023	954			0	H
*trnV*	1026	1097	72			2	H
*rrnL*	1120	2749	1630			22	H
*trnL2*	2775	2850	76			25	H
*nad1*	2852	3826	975	ATG	TAA	1	H
*trnI*	3831	3902	72			4	H
*trnQ*	3901	3971	71			–2	L
*trnM*	3974	4042	69			2	H
*nad2*	4043	5089	1047	ATG	TAG	0	H
*trnW*	5088	5158	71			–2	H
*trnA*	5161	5229	69			2	L
*trnN*	5231	5303	73			1	L
*trnC*	5337	5403	67			33	L
*trnY*	5403	5473	71			–1	L
*cox1*	5475	7025	1551	GTG	TAA	1	H
*trnS2*	7026	7096	71			0	L
*trnD*	7099	7170	72			2	H
*cox2*	7184	7874	691	ATG	T--	13	H
*trnK*	7875	7950	76			0	H
*atp8*	7952	8116	165	ATG	TAG	1	H
*atp6*	8110	8793	684	ATG	TAA	–7	H
*cox3*	8793	9578	786	ATG	TAA	–1	H
*trnG*	9578	9649	72			–1	H
*nad3*	9650	10,000	351	ATG	TAG	0	H
*trnR*	9999	10,068	70			–2	H
*nad4L*	10,069	10,365	297	ATG	TAA	0	H
*nad4*	10,359	11,739	1381	ATG	T--	–7	H
*trnH*	11,740	11,808	69			0	H
*trnS1*	11,809	11,876	68			0	H
*trnL1*	11,878	11,950	73			1	H
*nad5*	11,954	13,777	1824	ATG	TAA	3	H
*nad6*	13,774	14,295	522	ATG	TAA	–4	L
*trnE*	14,296	14,364	69			0	L
*cytb*	14,369	15,509	1141	ATG	T--	4	H
*trnT*	15,510	15,581	72			0	H
*trnP*	15,581	15,650	70			–1	L
D-loop	15,651	16,589	939			0	H

**Table 6:** Nucleotide composition and skewness of *S. curvilabiatus*.

Region	Size (bp)	T(U) (%)	C (%)	A (%)	G (%)	AT (%)	GC (%)	GT (%)	ATskew (%)	GCskew (%)
PCGs	11,412	32.8	22.2	21.6	23.6	54.3	45.8	56.3	−0.210	0.026
*atp6*	684	27.8	27.3	30.3	14.6	58.1	41.9	42.4	0.043	−0.303
*atp8*	165	27.3	26.7	33.3	12.7	60.6	39.4	40.0	0.1.00	−0.354
*cox1*	1551	29.4	25.8	26.1	18.7	55.5	44.5	48.1	−0.059	−0.159
*cox2*	691	28.2	24.9	30.4	16.5	58.6	41.4	44.7	0.037	−0.203
*cox3*	786	26.7	28.1	27.6	17.6	54.3	45.7	44.3	0.016	−0.231
*cytb*	1141	28.7	27.9	26.3	17.2	55.0	45.1	45.9	−0.043	−0.237
*nad1*	975	25.1	30.8	25.5	18.6	50.6	49.4	43.7	0.008	−0.247
*nad2*	1047	22.7	31.4	28.9	16.9	51.6	48.3	39.6	0.120	−0.300
*nad3*	351	27.6	29.1	29.6	13.7	57.2	42.8	41.3	0.035	−0.360
*nad4*	1381	26.1	28.7	28.8	16.4	54.9	45.1	42.5	0.050	−0.274
*nad4L*	297	26.9	31.0	25.6	16.5	52.5	47.5	43.4	−0.026	−0.305
*nad5*	1824	25.1	29.5	30.6	14.8	55.7	44.3	39.9	0.100	−0.332
*nad6*	522	38.9	15.9	14.8	30.5	53.7	46.4	69.4	−0.450	0.314
*rrnL*	1630	19.7	23.4	36.5	20.4	56.2	43.8	40.1	0.299	−0.067
*rrnS*	954	20.8	25.9	30.8	22.5	51.6	48.4	43.3	0.195	−0.069
rRNAs	2584	20.1	24.3	34.4	21.2	54.5	45.5	41.3	0.263	−0.068
tRNAs	1562	27.6	20.6	27.5	24.4	55.1	44.9	51.9	−0.003	0.084
Full genome	16,589	25.4	27.1	30.1	17.5	55.5	44.6	42.9	0.085	−0.214
D-Loop	939	28.8	23.4	30.6	17.2	59.4	40.6	46.0	0.030	−0.152

## Data Availability

The data that support the findings of this study are freely available in SRA and GenBank of NCBI (https://www.ncbi.nlm.nih.gov/). The raw data accession number is SRR32816543. The complete mitochondrial genome sequence accession number is PV287704.

## References

[1] Liu Y.C., Wei C. (2022). Age and growth characteristics of *Schizothorax curilabiatus* in the Chayu River, Tibet. Hubei Agric. Sci..

[2] Wang J., Liu F., Gong Z., Lin P.C., Liu H.Z., Gao X. (2016). Length–weight relationships of five endemic fish species from the lower Yarlung Zangbo River, Tibet, China. J. Appl. Ichthyol..

[3] Zhang J.L., Huang J.Q., Fang C., Xu T.Q., Wang K.F. (2021). Study on the biological characteristics of *Schizothorax curvilabiatus*. Hebei Fish..

[4] Wang J., Zhang F.B., Hu H.M., Gong Z., Cao W.X., Lin P.C. (2022). Characteristics of age and growth *Schizothorax curvilabiatus* in the lower reaches of the Yarlung Zangbo River. Acta Hydrobiol. Sin..

[5] Cao C.Y., Miao J., Xie Q.Q., Sun J.B., Cheng H., Zhang Z.Y., Wu F., Liu S., Ye X.W., Zhang Z. (2024). The first near-complete genome assembly of pig: Enabling more accurate genetic research. bioRxiv.

[6] Lian Q.C., Huettel B., Walkemeier B., Mayjonade B., Lopez-Roques C., Gil L., Roux F., Schneeberger K., Mercier R. (2024). A pan-genome of 69 *Arabidopsis thaliana* accessions reveals a conserved genome structure throughout the global species range. Nat. Genet..

[7] Oliveira J., Yildirir G., Corradi N. (2024). From chaos comes order: Genetics and genome biology of Arbuscular Mycorrhizal Fungi. Annu. Rev. Microbiol..

[8] Gao L.X., Chen S.Y., Feng T.B., Liu B.J., Liu Y.F., Shen H.D., Huang W.H., Liang X.D. (2024). Whole-genome analysis and microsatellite distribution of *Ilisha elongata*. Fish. Sci. Technol. Inf..

[9] Gu H.R., Wang S., Yang C.H., Tao M., Wang Z.J., Liu S.J. (2024). Global cooling and hot springs may have induced autotetraploidy and autohexaploidy in *Schizothorax* ancestors, and its implications for polyploid breeding. Aquaculture.

[10] Liu Y.C., Chen F., Wei C., Li J.C. (2022). Correlation and path analysis of morphological traits on body mass of juvenile *Schizothorax Curvilabiatus* at Two Sizes. Anhui Agric. Sci. Bull..

[11] Zhang C., Li K., Su Q. Genetic diversity and population structure of *Schizothorax curvilabiatus* in the lower reaches of Yarlung Zangbo River. J. Fish. Sci. China.

[12] Jin H.L., Li L., Tan D.L., Wu S., Wang N.M., Jin X., Ma B. (2022). Nutritional composition in muscle of *Schizothorax curvilabiatus*. Biot. Resour..

[13] Ma H.X. (2019). Development of SNP Markers and Population Genetics Analysis of Schizothorax curvilabiatus Based on SLAF-seq Technology.

[14] Liu M.D., Zhu F.Y., Zhu T.B., Li L., Wang L., Liu X.J., Zhu R., Liu F., Cen X., Hu F.F. (2025). Status of aquatic organisms resources and their environments in Xizang (2017—2021). J. Fish. China.

[15] Wang W.L., Zhang L.H., Pan Y.Z., Lhamo T., Zhang C., Li B.H. (2017). The complete mitochondrial genome of the *Schizothorax curilabiatus* (Cypriniformes: Cyprinidae). Mitochondrial DNA Part B.

[16] Mason A.S. (2015). SSR genotyping. Plant Genotyping: Methods Protocols.

[17] Sun N.R., Chen J.S., Wang Y.Q., Hussain I., Lei N., Ma X.Y., Li W.Q., Liu K.W., Yu H.R., Zhao K. (2024). Development and utility of SSR markers based on *Brassica* sp. whole-genome in triangle of U. Front. Plant Sci..

[18] Hu L., Wang J.C., Wang X.Y., Zhang D.Y., Sun Y.X., Lu T., Shi W. (2024). Development of SSR markers and evaluation of genetic diversity of endangered plant *Saussurea involucrata*. Biomolecules.

[19] Zhao M.Q., Shu G.P., Hu Y.H., Cao G.Q., Wang Y.B. (2023). Pattern and variation in simple sequence repeat (SSR) at different genomic regions and its implications to maize evolution and breeding. BMC Genom..

[20] Ozdemir D., Bener L., Akcay E.T. Optimizing Genomic DNA Extraction from Avian Feathers: A Modified Phenol–Chloroform Approach for Enhanced Efficiency and Cost-Effectiveness. Biochem. Genet..

[21] Chen S.F., Zhou Y.Q., Chen Y.R., Gu J. (2018). fastp: An ultra-fast all-in-one FASTQ preprocessor. Bioinformatics.

[22] Brown J., Pirrung M., McCue L.A. (2017). FQC Dashboard: Integrates FastQC results into a web-based; interactive; and extensible FASTQ quality control tool. Bioinformatics.

[23] Luleci H.B., Yuka S.A., Yilmaz A. Efficient Storage and Analysis of Genomic Data: A k-mer Frequency Mapping and Image Representation Method. Interdiscip. Sci. Comput. Life Sci..

[24] Luo R.B., Liu B.H., Xie Y.L., Li Z.Y., Huang W.H., Yuan J.Y., He G.Z., Chen Y.X., Pan Q., Liu Y.J. (2012). SOAPdenovo2:an empirically improved memory-efficient short-read de novo assembler. GigaScience.

[25] Kõressaar T., Lepamets M., Kaplinski L., Raime K., Andreson R., Remm M. (2018). Primer3_masker: Integrating masking of template sequence with primer design software. Bioinformatics.

[26] Chen N.S. (2004). Using Repeat Masker to identify repetitive elements in genomic sequences. Curr. Protoc. Bioinform..

[27] Flynn J.M., Hubley R., Goubert C., Rosen J., Clark A.G., Feschotte C., Smit A.F. (2020). RepeatModeler2 for automated genomic discovery of transposable element families. Proc. Natl. Acad. Sci. USA.

[28] Altschul S.F., Madden T.L., Schäffer A.A., Zhang J.H., Zhang Z., Miller W., Lipman D.J. (1997). Gapped BLAST and PSI-BLAST: A new generation of protein database search programs. Nucleic Acids Res..

[29] Dierckxsens N., Mardulyn P., Smits G. (2017). NOVOPlasty: De novo assembly of organelle genomes from whole genome data. Nucleic Acids Res..

[30] Bernt M., Donath A., Jühling F., Externbrink F., Florentz C., Fritzsch G., Pütz J., Middendorf M., Stadler P.F. (2013). MITOS: Improved de novo metazoan mitochondrial genome annotation. Mol. Phylogenetics Evol..

[31] Hall T., Biosciences I., Carlsbad C. (2011). BioEdit: An important software for molecular biology. GERF Bull. Biosci..

[32] Grant J.R., Stothard P. (2008). The CGView Server: A comparative genomics tool for circular genomes. Nucleic Acids Res..

[33] Zhang D., Gao F.L., Jakovlić I., Zou H., Zhang J., Li W.X., Wang G.T. (2020). PhyloSuite: An integrated and scalable desktop platform for streamlined molecular sequence data management and evolutionary phylogenetics studies. Mol. Ecol. Resour..

[34] Chan P.P., Lin B.Y., Mak A.J., Lowe T.M. (2021). tRNAscan-SE 2.0: Improved detection and functional classification of transfer RNA genes. Nucleic Acids Res..

[35] Katoh K., Kuma K., Toh H., Miyata T. (2005). MAFFT version 5: Improvement in accuracy of multiple sequence alignment. Nucleic Acids Res..

[36] Talavera G., Castresana J. (2007). Improvement of phylogenies after removing divergent and ambiguously aligned blocks from protein sequence alignments. Syst. Biol..

[37] Tamura K., Stecher G., Kumar S. (2021). MEGA11: Molecular evolutionary genetics analysis version 11. Mol. Biol. Evol..

[38] Letunic I., Bork P. (2021). Interactive Tree Of Life (iTOL) v5: An online tool for phylogenetic tree display and annotation. Nucleic Acids Res..

[39] Aparicio S., Chapman J., Stupka E., Putnam N., Chia J., Dehal P., Christoffels A., Rash S., Hoon S., Smit A. (2002). Whole-genome shotgun assembly and analysis of the genome of *Fugu rubripes*. Science.

[40] Liu Y.G., Xu P., Xu J.M., Huang Y., Liu Y.X., Fang H., Hu Y.C., You X.X., Bian C., Sun M. (2017). China is initiating the aquatic 10-100-1000 genomics program. Life Sci..

[41] Fan G.Y., Song Y., Yang L.D., Huang X.Y., Zhang S.Y., Zhang M.Q., Yang X.W., Chang Y., Zhang H., Li Y.X. (2020). Initial data release and announcement of the 10,000 Fish Genomes Project (Fish10K). GigaScience.

[42] Woods I.G., Wilson C., Friedlander B., Chang P., Reyes D.K., Nix R., Kelly P.D., Chu F., Postlethwait J.H., Talbot W.S. (2005). The zebrafish gene map defines ancestral vertebrate chromosomes. Genome Res..

[43] Dong Z.D., Wang J.M., Chen G.Z., Guo Y.S., Zhao N., Wang Z.D., Zhang B. (2024). A high-quality chromosome-level genome assembly of the Chinese medaka *Oryzias sinensis*. Sci. Data.

[44] Xu P., Zhang X.F., Wang X.M., Li J.T., Liu G.M., Kuang Y.Y., Xu J., Zheng X.H., Ren L.F., Wang G.L. (2014). Genome sequence and genetic diversity of the common carp; *Cyprinus carpio*. Nat. Genet..

[45] Wu C.S., Ma Z.Y., Zheng G.D., Zou S.M., Zhang X.J., Zhang Y.A. (2022). Chromosome-level genome assembly of grass carp (*Ctenopharyngodon idella*) provides insights into its genome evolution. BMC Genom..

[46] Warren W.C., Boggs T.E., Borowsky R., Carlson B.M., Ferrufino E., Gross J.B., Hillier L., Hu Z.L., Keene A.C., Kenzior A. (2021). A chromosome-level genome of *Astyanax mexicanus* surface fish for comparing population-specific genetic differences contributing to trait evolution. Nat. Commun..

[47] Zhou C.W., Zhou Y., Xu L.H., Liu F., Lei L., Gao H., Li J.T., Fu S.X., Duan Y.T., Tan Y.G. Chromosome-level genome assembly and population genomic analysis provide insights into the genetic diversity and adaption of *Schizopygopsis younghusbandi* on the Tibetan Plateau. Integr. Zool..

[48] Liu M.D., Ma B., Zhang C., Tang T., Liu S.P., Duan X.B., Li L., Zhu F.Y., Wang N.M., Chen D.Q. (2020). Distribution pattern and environmental impact factors of Schizothoracinae fishes in the rivers of Tibet: The case of Nujiang River and Yalu Zangbo River. Ecol. Environ. Sci..

[49] Niu Z.G., Jing Y., Zhang D.Q., Zhang B. (2024). An overview and the outlook for wetland ecosystems in the Qinghai-Tibetan Plateau under climate change. Clim. Change Res..

[50] Xiao S.J., Mou Z.B., Fan D.D., Zhou H., Zou M., Zou Y., Zhou C.W., Yang R.B., Liu J.Q., Zhu S.L. (2020). Genome of tetraploid fish *Schizothorax o’connori* provides insights into early re-diploidization and high-altitude adaptation. Iscience.

[51] Liang X.G., Wang W.H., Huang J.R., Luo M.F., Wang N., Sun C.Y., Lu J.G. (2024). A chromosome-level genome assembly of big-barbel schizothorcin; *Schizothorax macropogon*. Sci. Data.

[52] Liu H.P., Xiao S.J., Wu N., Wang D., Liu Y.C., Zhou C.W., Liu Q.Y., Yang R.B., Jiang W.K., Liang Q.Q. (2019). The sequence and de novo assembly of *Oxygymnocypris stewartii* genome. Sci. Data.

[53] Dodsworth S., Chase M.W., Leitch A.R. (2016). Is post-polyploidization diploidization the key to the evolutionary success of angiosperms?. Bot. J. Linn. Soc..

[54] Chen L., Li C.Y., Li B.J., Zhou X.F., Bai Y.L., Zou X.Q., Zhou Z.X., He Q., Chen B.H., Wang M. (2024). Evolutionary divergence of subgenomes in common carp provides insights into speciation and allopolyploid success. Fundam. Res..

[55] Ye H., Zhang Z.S., Zhou C.W., Zhu C.K., Yang Y.J., Xiang M.B., Zhou X.H., Zhou J., Luo H. (2018). De novo assembly of *Schizothorax waltoni* transcriptome to identify immune-related genes and microsatellite markers. RSC Adv..

[56] Ren Y.L. (2020). Genetic Diversity of Schizothorax biddulphi Based on SSR Markers and mtDNA Sequences.

[57] Nie Z.L., Ren Y.L., Zhang L.R., Ge R., Wei J. (2022). Analysis of population genetic diversity and genetic structure of *Schizothorax biddulphi* based on 20 newly developed SSR markers. Front. Genet..

[58] Luo H., Xiao S.J., Ye H., Zhang Z.S., Lv C.H., Zheng S.M., Wang Z.Y., Wang X.Q. (2016). Identification of immune-related genes and development of SSR/SNP markers from the spleen transcriptome of *Schizothorax prenanti*. PLoS ONE.

[59] Li X.H., Tang Y.T., Zhang R.Y., Tian F., Zhao K. (2020). Characterization and development of SSR markers of schizothoracine fish (Cypriniformes: Cyprinidae) based on SLAF-seq Technique. J. Appl. Ichthyol..

[60] Shen J.Y., Su T.H., Yu D.Q., Tan S.J., Zhang Y. (2024). Evolution by gene duplication: In the era of genomics. Hereditas.

[61] Jaggi K.E., Krak K., Štorchová H., Mandák B., Marcheschi A., Belyayev A., Jellen E.N., Sproul J., Jarvis D., Maughan P.J. (2025). A pangenome reveals LTR repeat dynamics as a major driver of genome evolution in *Chenopodium*. Plant Genome.

[62] Qi H.R. (2015). Chromosome Manipulation and Microsatellite Study of Natural Tetraploid Loach (Misgurnus anguillicaudatus).

[63] Kidwell M.G., Lisch D.R. (2000). Transposable elements and host genome evolution. Trends Ecol. Evol..

[64] Betancourt A.J., Wei K.H.C., Huang Y.H., Lee Y.C.G. (2024). Causes and consequences of varying transposable element activity: An evolutionary perspective. Annu. Rev. Genom. Hum. Genet..

[65] Liu H.P., Liu Q.Y., Chen Z.Q., Liu Y.C., Zhou C.W., Liang Q.Q., Ma C.X., Zhou J.S., Pan Y.Z., Chen M.Q. (2018). Draft genome of *Glyptosternon maculatum;* an endemic fish from Tibet Plateau. GigaScience.

[66] Zhou C.J., Hu B., Tang Y.T., Yang C.X., Ma W.W., Wang X., Liu R.Y., Yan X.M., Dong J., Wang X.F. (2021). The chromosome-level genome of *Triplophysa dalaica* (Cypriniformes: Cobitidae) provides insights into its survival in extremely alkaline environment. Genome Biol. Evol..

[67] Tian F., Liu S.J., Zhou B.Z., Tang Y.T., Zhang Y., Zhang C.F., Zhao K. (2022). Chromosome-level genome of Tibetan naked carp (*Gymnocypris przewalskii*) provides insights into Tibetan highland adaptation. DNA Res..

[68] Zhang X.M., Xiong D.M., Jian S.L., Jiang Y., Wang L.X. (2023). Chromosome-level genome assembly for Sichuan taimen (*hucho bleekeri*) reveals the extraordinary tandem repeat proportions and its persistent population shrinkage. bioRxiv.

[69] Rey O., Danchin E., Mirouze M., Loot C., Blanchet S. (2016). Adaptation to global change: A transposable element–epigenetics perspective. Trends Ecol. Evol..

[70] Schirrmacher V. (2020). Mitochondria at work: New insights into regulation and dysregulation of cellular energy supply and metabolism. Biomedicines.

[71] Vučković A., Freyer C., Wredenberg A., Hillen H.S. (2024). The molecular machinery for maturation of primary mtDNA transcripts. Hum. Mol. Genet..

[72] Ferreira T., Rodriguez S. (2024). Mitochondrial DNA: Inherent complexities relevant to genetic analyses. Genes.

[73] Liu L., Liu Q., Gao T.X. (2022). Genome-wide survey reveals the phylogenomic relationships of Chirolophis japonicus Herzenstein; 1890 (Stichaeidae; Perciformes). ZooKeys.

[74] Persi E., Wolf Y.I., Horn D., Ruppin E., Demichelis F., Gatenby R.A., Gillies R.J., Koonin E.V. (2021). Mutation–selection balance and compensatory mechanisms in tumour evolution. Nat. Rev. Genet..

[75] Zhu Y.X., Chen Y., Cheng Q.Q., Qiao H.Y., Chen W.M. (2013). The complete mitochondrial genome sequence of *Schizothorax macropogon* (Cypriniformes: Cyprinidae). Mitochondrial DNA.

[76] Chen Y., Cheng Q.Q., Qiao H.Y., Zhu Y.X., Chen W.M., Ren G.J. (2013). The complete mitochondrial genome sequence of *Schizothorax wangchiachii* (Cypriniformes: Cyprinidae). Mitochondrial DNA.

[77] Akhter G., Ahmed I., Ahmad S.M. (2024). Genomic analysis and phylogenetic characterization of Himalayan snow trout; *Schizothorax* esocinus based on mitochondrial protein-coding genes. Mol. Biol. Rep..

[78] Iyyappan S., Rather M.A., Ahmad I., Ahmad I. (2024). Comparative mitochondrial genomics analysis of selected species of Schizothoracinae sub family to explore the differences at mitochondrial DNA level. Comput. Biol. Chem..

[79] Liu Y.P., Hu J.Y., Ning Z.J., Xiao P.Y., Yang T.Y. (2023). Mitochondrial genome sequence characteristics and phylogenetic analysis of *Schizothorax argentatus*. Chin. J. Biotechnol..

[80] Shibata T., Ikawa S., Iwasaki W., Sasanuma H., Masai H., Hirota K. (2024). Homology recognition without double-stranded DNA-strand separation in D-loop formation by RecA. Nucleic Acids Res..

[81] Liu Y.Q., Zhao S., Chen X.L., Bian Y.H., Cao Y.Z., Xu P.W., Zhang C.M., Zhang J.T., Zhao S.G., Zhao H. (2023). Variations in mitochondrial DNA coding and D-loop region are associated with early embryonic development defects in infertile women. Hum. Genet..

[82] Song D., Peng D., Cheng Q.Q. (2023). Phylogenetic and adaptive evolution analysis of Schizothoracinae fish and the taxonomic status of *Gymnocypris chilianensis*. J. Fish. Sci. China.

[83] Qin Q., Chen L., Zhang F.B., Xu J.H.Y., Zeng Y. (2024). Characterization of the complete mitochondrial genome of *Schizothorax kozlovi*(Cypriniformes; Cyprinidae; *Schizothorax*) and Insights intothe Phylogenetic Relationships of *Schizothorax*. Animals.

[84] Rozimov A., Wang Y.F., Wang M., Zou M., Sobirov J., Karimov E., Kholmatov B., Freyhof J., Namozov S., Wang C. (2025). Mitochondrial genome insights into the phylogenetics and biogeographic evolution of snow trout (Cyprinidae; *Schizothorax*) in the Tien Shan Mountains. Zoosystematics Evol..

[85] Toews D.P.L., Brelsford A. (2012). The biogeography of mitochondrial and nuclear discordance in animals. Mol. Ecol..

[86] Alix K., Gérard P.R., Schwarzacher T., Heslop-Harrison J.S.P. (2017). Polyploidy and interspecific hybridization: Partners for adaptation, speciation and evolution in plants. Ann. Bot..

[87] Gao X., Zhang H., Cui J.L., Yan X.J., Zhang X.Y., Luo M.X., Tang C.C., Ren L., Liu S.J. (2021). Interactions between mitochondrial and nuclear genomes and co-regulation of mitochondrial and nuclear gene expression in reciprocal intergeneric hybrids between *Carassius auratus* red var.× *Cyprinus carpio* L. Reprod. Breed..

